# Role of Periostin Expression in Non-Small Cell Lung Cancer: Periostin Silencing Inhibits the Migration and Invasion of Lung Cancer Cells via Regulation of MMP-2 Expression

**DOI:** 10.3390/ijms23031240

**Published:** 2022-01-22

**Authors:** Katarzyna Ratajczak-Wielgomas, Alicja Kmiecik, Piotr Dziegiel

**Affiliations:** 1 Division of Histology and Embryology, Department of Human Morphology and Embryology, Wroclaw Medical University, 50-368 Wroclaw, Poland; alicja.kmiecik@umw.edu.pl (A.K.); piotr.dziegiel@umw.edu.pl (P.D.); 2 Department of Human Biology, Faculty of Physiotherapy, University School of Physical Education, 51-612 Wroclaw, Poland

**Keywords:** periostin, non-small cell lung carcinoma, invasion, matrix metalloproteinase-2, remodeling, extracellular matrix

## Abstract

The involvement of periostin (POSTN) in non-small-cell lung cancer (NSCLC) migration, invasion, and its underlying mechanisms has not been well established. The present study aims to determine epithelial POSTN expression in NSCLC and to assess associations with clinicopathological factors and prognosis as well as to explore the effects of POSTN knockdown on tumor microenvironment and the migration and invasion of lung cancer cells. Immunohistochemistry was used to evaluate epithelial POSTN expression in NSCLC. POSTN mRNA expression in the dissected lung cancer cells was confirmed by laser capture microdissection and real-time PCR. A549 cells were used for transfecting shRNA-POSTN lentiviral particles. Wound healing and Transwell invasion assays were used to assess the migratory and invasive abilities of A549 cells transfected with POSTN-specific short hairpin (sh)RNA. The results demonstrated significantly higher cytoplasmic POSTN expression in the whole NSCLC group compared to non-malignant lung tissue (NMLT). POSTN expression in cancer cells may be considered to be an independent prognostic factor for survival in NSCLC. POSTN knockdown significantly inhibited A549 cell migration and invasion capabilities in vitro. The activity and the expression level of matrix metalloproteinase-2 (MMP-2) were significantly decreased in A549.shRNA compared to control cells. In summary, POSTN may regulate lung cancer cell invasiveness by modulating the expression of MMP-2 and may represent a potential target for novel therapeutic intervention for NSCLC.

## 1. Introduction

Lung cancer, i.e., bronchogenic malignant neoplasms arising from airway epithelioma, is one of the most common malignancies, and 5-year survival rates range from 4% to 17%, depending on the stage of the disease at diagnosis [1,2]. Worldwide, over 1.5 million people annually develop non-small cell lung cancer (NSCLC), which accounts for 80–85% of all lung cancer cases [3,4]. Improvements in the knowledge of molecular alterations and their functional significance have the prospective to influence lung cancer diagnosis, prognostication, and treatment [5]. Tumor invasion and metastasis are one of the major causes of lung cancer-associated mortality. Therefore, to improve lung cancer treatment, the mechanism underlying lung cancer metastasis should be completely understood so that the establishment of methods that suppress tumor metastasis can be facilitated [6].

The molecular mechanisms underlying cancer cell invasion and migration are complex. The initial events are related to the proteolytic degradation of the extracellular matrix (ECM), which provides biochemical and mechanical barriers to cancer cell migration [7]. ECM degradation requires the expression and activity of matrix metalloproteinases (MMPs), which are known to play a major role in lung cancer by favoring the invasion of cancer cells [7,8]. Among the MMPs, matrix metalloproteinase -2 (MMP-2) activation is related to tumor progression and invasion [7,8]. Therefore, the inhibition of the MMP-2 expression regulatory pathway is an important therapeutic strategy for preventing lung cancer metastasis.

Periostin (POSTN), a secreted matrix N-glycoprotein that lacks a transmembrane domain, is a multimodular protein with a signal peptide (SP) that is crucial for secretion, a cysteine-rich domain in the EMILIN family (EMI domain) involved in the formation of multimers through cysteine disulfide bonds [9], a tandem of four homologous FAS1 domains (FAS1) that interact with integrins (αvβ3, αvβ5, α6β4) [10], and a C-terminal region (CTRL) regulating the cell–matrix organization and interactions by binding the ECM proteins such as collagens, fibronectin, tenascin C, or heparin [9,11]. These properties make POSTN a key player in the regulation of cell behavior and the organization of the ECM. POSTN is a protein expressed in various normal human tissues and plays a role in many normal physiological processes. It has been demonstrated that POSTN is involved in the physiological process of epithelial–mesenchymal transition (EMT). This glycoprotein plays a significant role in the fibrillogenesis of collagen [12] and the wound healing process [13]. Additionally, POSTN is also associated with pathological processes, including the development of cardiovascular diseases, inflammation, asthma, and tumorigenesis [14]. The mechanism by which POSTN interacts with tumors has not been fully understood. Most analyses have indicated that POSTN can be involved in regulating the ECM network [14]. It interacts with other ECM proteins to form an extracellular environment where cells can interact with each other to promote both growth and survival [15]. POSTN has also been confirmed to regulate the development of several types of human cancers by binding to the integrins to activate the Akt/PKB and FAK-mediated signaling pathways [16,17]. POSTN-activated signaling pathways promote cellular survival, motility, and adhesion, which are crucial in tumor growth, angiogenesis, invasion, and metastasis. [17]. Additionally, it induces neovascularization and supports tumor growth by inducing vascular endothelial growth factor receptor expression in vascular endothelial cells, also stimulating the survival of vascular endothelial cells through the Akt pathway [18,19,20]. Currently, many studies have indicated that the overexpression of POSTN is connected with tumor progression. However, others have reported that POSTN might inhibit the invasion and metastasis of bladder cancer cells [21]. Furthermore, Kanno et al. [22] demonstrated that POSTN had dual effects: the promotion and inhibition of pancreatic cancer. These results indicate the variable biological effects of POSTN in different tissues, which suggest the need for further studies on its complex and multi-aspect functions [23].

The aim of the study is to determine the cytoplasmic expression of POSTN in NSCLC as well as in some histological subtypes such as adenocarcinoma (AC) and squamous cell carcinoma (SCC) in relation to clinicopathological data and prognosis. Furthermore, to study the biological role of POSTN in the progression of NSCLC, we used shRNA to silence the expression of POSTN in lung cancer cells (the loss of function phenotype model). Therefore, this study aims to analyze the effect of POSTN-silencing on migratory and invasiveness of lung carcinoma A549 cells and the expression of tumor microenvironment factor MMP-2 and integrin-signaling-pathway-related proteins.

## 2. Results

### 2.1. Examination of POSTN Expression Level and Its Association with Clinicopathological Parameters of Patients

**Figure 1:**
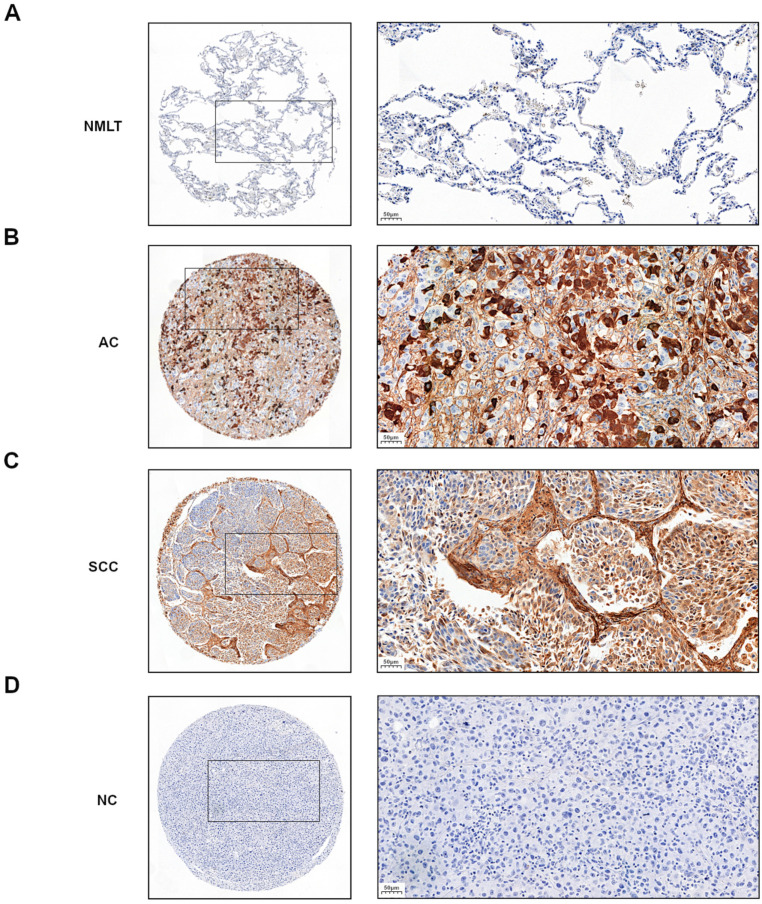
Representative immunohistochemical images with cytoplasmic periostin (POSTN) expression in (**A**) non-malignant lung tissue (NMLT) and non-small cell lung carcinoma (NSCLC) subtypes: (**B**) adenocarcinoma (AC) and (**C**) squamous cell carcinoma (SCC). (**D**) negative control (NC). Magnification 50×, 200×. Bar = 200 µm and 50 µm, respectively.

In the analyzed NSCLC cases and the particular subtypes such as AC and SCC, positive cytoplasmic (90.9%) immunohistochemistry (IHC) expression of POSTN was demonstrated (Figure 1B,C). The mean value of POSTN expression (IHC) was 3.9. Sections with a score of 0–3 pts were considered ‘low’, whereas those with 4–12 pts were ‘high’.

**Figure 2:**
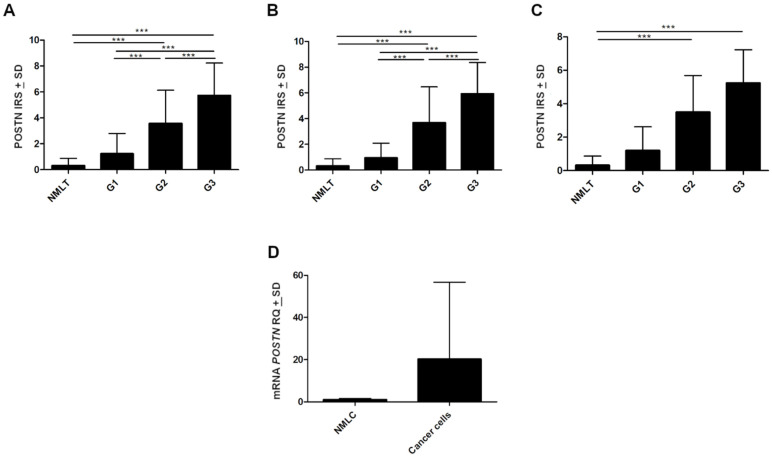
Immunohistochemical evaluation of periostin (POSTN) expression level according to the grade of malignancy (G) in (**A**) non-small cell lung carcinoma (NSCLC), (**B**) adenocarcinoma (AC), and (**C**) squamous cell carcinoma (SCC) compared to non-malignant lung tissue (NMLT), (*** *p* < 0.001). (**D**) The expression level of mRNA *POSTN* in microdissected (LCM) cancer cells compared to their expression in non-malignant lung cells (NMLC). IRS, immunoreactive score; RQ, relative quantification.

Cytoplasmic POSTN expressions were significantly higher in NSCLC as well as in SCC and AC subtypes compared to non-malignant lung tissue (NMLT) (**** *p* < 0.0001, respectively; Mann–Whitney *U*-test); (Figure 1A–C and Figure 2A–C). These results were also confirmed by molecular studies using laser capture microdissection (LCM). We noticed a markedly higher level of *POSTN* mRNA expression in microdissected cancer cells compared to non-malignant lung cells (NMLC) (Figure 2D). Furthermore, statistical analysis demonstrated an increasing level of POSTN (IHC) with an increasing malignancy grade (G) of the tumor in the whole cohort, as well as in AC cases. A significant difference was noted between G1vs G2 and G1vs G3 (*** *p* < 0.001 in both cases, Mann–Whitney *U*-test); (Figure 2A,B). In SCC cases, we noted an increasing level of POSTN expression with an increasing malignancy grade. However, the differences did not reach the threshold of statistical significance (*p* > 0.05, Mann–Whitney *U*-test); (Figure 2C).

**Figure 3:**
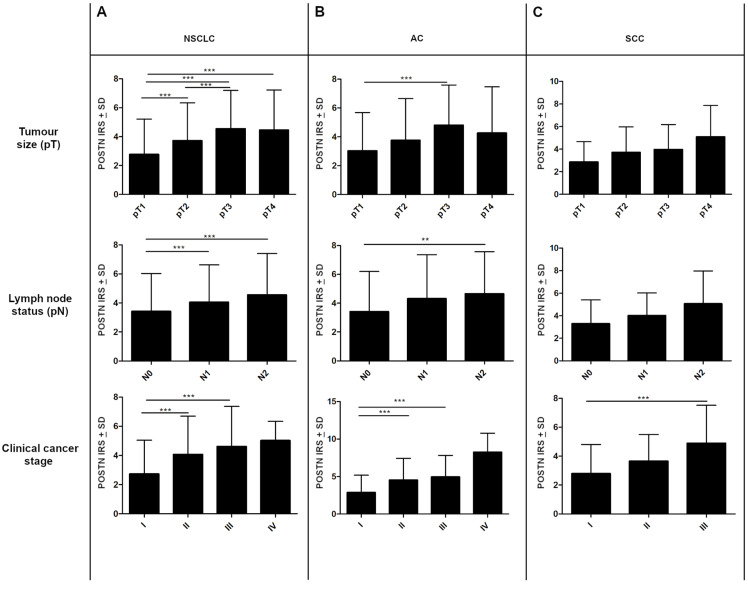
The expression level of periostin (POSTN) in regard to patients’ clinicopathological factors. Comparison of expression levels of POSTN in cancer cells of non-small cell lung carcinoma (NSCLC) with respect to tumor size (pT), lymph node status (pN), and clinical cancer stage in the (**A**) whole study cohort as well as in individual histological types, i.e., (**B**) adenocarcinoma (AC) and (**C**) squamous cell carcinoma (SCC); (** *p* < 0.005, *** *p* < 0.001). IRS, immunoreactive score.

Statistically, differences in POSTN expression in cancer cells were also noted regarding lymph node status. Significant differences were found between N0 vs. N1 and N0 vs. N2 in the whole study cohort (*** *p* < 0.001, respectively; Mann–Whitney *U*-test); (Figure 3A). In the AC group, statistically significant differences were noted only between N0 and N2 (** *p* < 0.005 Mann–Whitney *U*-test); (Figure 3B). However, in the SCC group, changes in POSTN expression in relation to the pN status were statistically insignificant (*p* > 0.05, Mann–Whitney *U*-test); (Figure 3C).

The expression level of POSTN also increased in higher tumor sizes (pT). We observed significantly higher cytoplasmic POSTN expression in pT3-T4 cases compared to pT1-T2 cases in the whole NSCLC group and the AC group (*** *p* < 0.001, respectively; Mann–Whitney *U*-test); (Figure 3A,B). However, in the SCC group, no statistically differences were shown in POSTN expression concerning pT (*p* > 0.05; Mann–Whitney *U*-test); (Figure 3C). In addition, compared to less advanced tumors (I, II), tumors in the advanced disease stage (III, IV) were characterized by higher cytoplasmic POSTN expression. A significant difference was noted between stages I vs. II and I vs. III in the whole NSCLC group, as well as in the AC group, (*** *p* < 0.001, respectively; Mann–Whitney *U*-test); (Figure 3A,B). In SCC cases, statistically significant differences were noted only between stages I and III (*** *p* < 0.001, respectively; Mann–Whitney *U*-test); (Figure 3C). Significant associations also were noted (in the whole cohort, AC) between cytoplasmic POSTN expression and gender as well as the smoking status (** *p* < 0.005; Mann–Whitney *U*-test).

### 2.2. Comparison of POSTN with MMP-2 Expression Level

**Figure 4:**
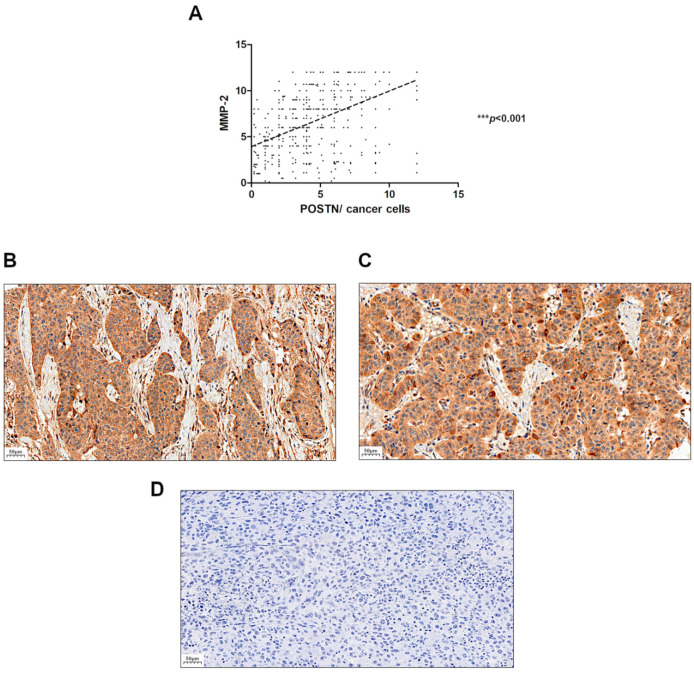
Correlation of periostin (POSTN) expression level with matrix metalloproteinase-2 (MMP-2) expression level in (**A**) non-small cell lung carcinoma (NSCLC), (r = 0.5262, *** *p* < 0.001). Immunohistochemical images with cytoplasmic MMP-2 expression in the tissue of two non-small cell lung carcinoma (NSCLC) subtypes: (**B**) squamous cell cancer (SCC) and (**C**) adenocarcinoma (AC). (**D**) negative control (NC). Magnification 200×. Bar = 50 µm.

We assessed the relationship between the expression level of POSTN and MMP-2 in NSCLC and found a positive significant correlation of POSTN expression level with MMP-2 in tumor cells (r = 0.5262, *** *p* < 0.001) (Figure 4).

### 2.3. Associations between Cytoplasmic and Stromal Expression of POSTN in NSCLC

**Figure 5:**
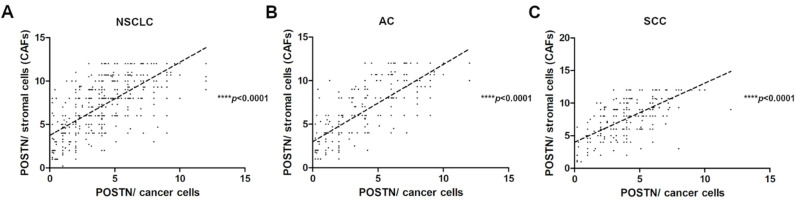
Correlations of periostin (POSTN) expression levels in the cytoplasm of cancer cells with POSTN expression in stromal cells (CAFs) in (**A**) non-small cell lung carcinoma (NSCLC) (r = 0.7085, **** *p* < 0.0001), (**B**) adenocarcinoma (AC), (r = 0.7897, **** *p* < 0.0001) and (**C**) squamous cell carcinoma (SCC) (r = 0.6607, **** *p* < 0.0001).

The correlation analysis showed a strong positive significant (r = 0.7085, **** *p* < 0.0001) correlation of POSTN expression in the cytoplasm of cancer cells with POSTN expression in tumor stromal cells (CAFs) in the whole cohort (Figure 5A). The results of POSTN expression in the stromal cells (CAFs) were used from previously conducted and published studies [24]. Furthermore, we also noticed a strong positive significant correlation between epithelial POSTN expression and the level of POSTN expression in stromal cells in AC and SCC subtypes (r = 0.7897, r = 0.6607, **** *p* < 0.0001, respectively) (Figure 5B,C).

### 2.4. Survival Analysis

**Figure 6:**
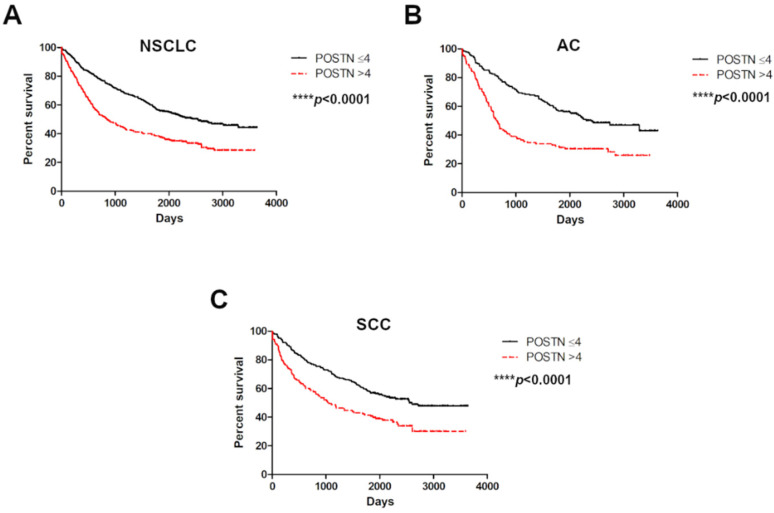
Kaplan–Meier overall survival curves in regard to cytoplasmic POSTN expression in (**A**) non-small cell lung carcinoma (NSCLC), (*N*= 715) and non-small cell lung carcinoma (NSCLC) subtypes: (**B**) adenocarcinoma (AC), (*N*= 298) and (**C**) squamous cell carcinoma (SCC), (*N*= 370). Analysis using the Mantel–Cox test. **** *p* < 0.0001. Cut-off points for the analysis were estimated based on the median.

The analysis related to the survival of the NSCLC cases demonstrated that patients with high cytoplasmic expression of POSTN in the tumor cells lived significantly shorter lives than patients with low POSTN expression in the whole NSCLC group (**** *p* < 0.0001) as well as with the particular histological types, such as AC and SCC (**** *p* < 0.0001); (Figure 6A–C).

Moreover, univariate and multivariate Cox survival analyses demonstrated that POSTN expression, pT, and pN were independent prognostic factors in NSCLC and AC cases (Table 1). A similar relationship was found in SCC cases analyzed separately. Multivariate Cox survival analyses showed that in the SCC group, POSTN expression, age, and pT were independent prognostic factors (Table 1).

### 2.5. The Expression of POSTN in Lung Cancer Cell Lines

**Figure 7:**
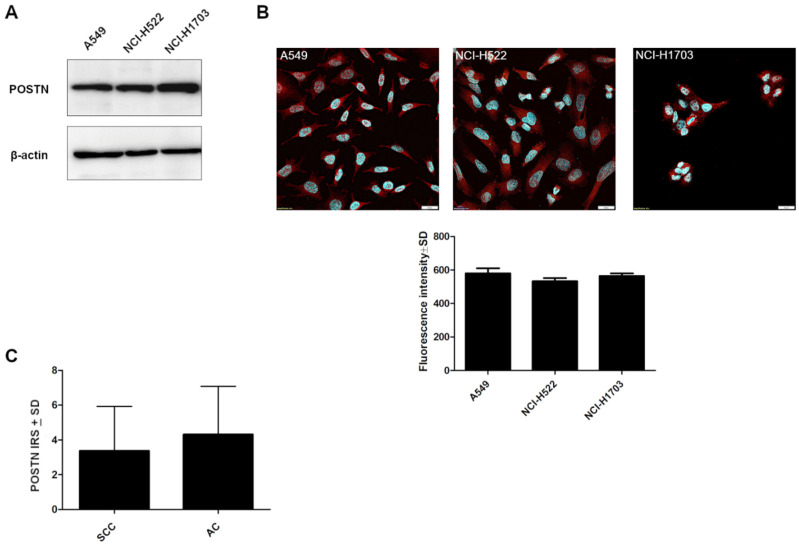
Characteristics of lung cancer cell lines according to periostin (POSTN) expression. (**A**) Comparison of POSTN expression levels detected by Western blot and (**B**) by confocal microscopy (fluorescence intensity) in different lung cancer cell lines (A549, NCI-H522, NCI-H1703), (*p* > 0.05). Objective 60×/1.40 Oil; pinhole airy 1.25. Bar = 20 µm. (**C**) Immunohistochemical expression level of periostin (POSTN) in the tissue of particular non-small cell lung carcinoma (NSCLC) subtypes: squamous cell carcinoma (SCC) and adenocarcinoma (AC) (*p* > 0.05). IRS, immunoreactive score.

To create a specific loss-of-function cellular model using shRNA, we screened the cultured lung cancer cell lines (NCI-H1703, A549, NCI-H522) for the expression of POSTN. Western blot and fluorescence analysis showed a comparable level of POSTN in the cell lines (Figure 7A,B).

A549 cells were chosen to continue the research on POSTN silencing because the A549 cell line is derived from highly malignant AC as opposed to the NCI-H522 cell line from stage II AC and NCI-1703 cell lines, which was derived from stage I SCC. Moreover, based on IHC levels, we showed a slight upward trend of cytoplasmic POSTN expression in the AC subtypes compared to the SCC type (Figure 7C, *p* > 0.05).

**Figure 8:**
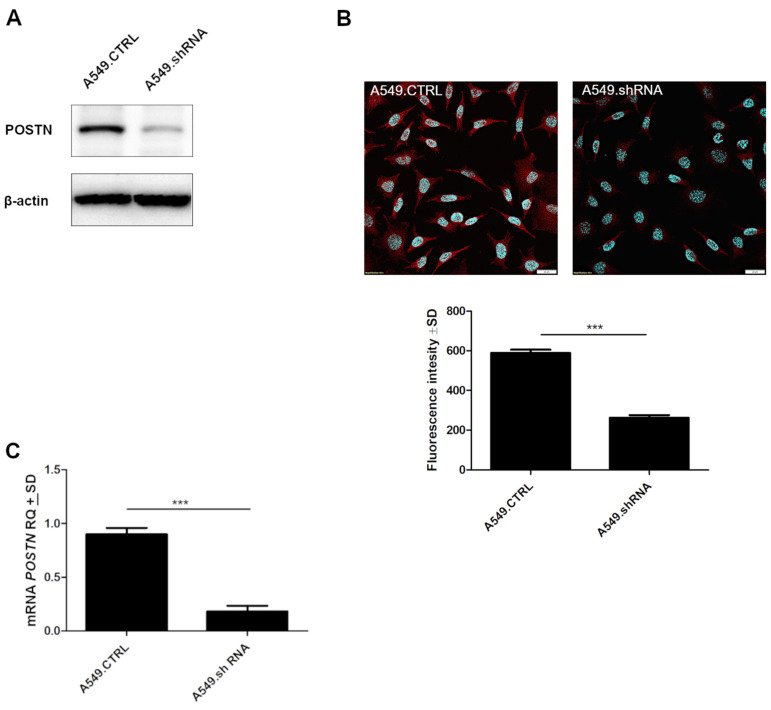
The efficacy of shRNA lentiviral particles transfection. (**A**) Silencing of periostin (POSTN) expression was confirmed by Western blot analysis. β-actin was used as an internal control. (**B**) Confocal images and fluorescence analysis showing the expression of POSTN in control A549.CTRL cells and A549.shRNA cells (*** *p* < 0.001). Objective 60×/1.40 Oil; pinhole airy 1.25. Bar = 20 µm. (**C**) The expression of *POSTN* mRNA in control cells (A549.CTRL) and the cells representing the loss-of-function phenotype (A549.shRNA). Relative expression (RQ) of the *POSTN* gene was normalized against the expression of ACTB (*** *p* < 0.001). RQ, relative quantification.

A549 cells were transduced with the expression vector, and the resulting cell population, resistant to puromycin, was screened for the presence of POSTN at the mRNA and protein levels. The generated cells with silenced expression of POSTN were termed A549.shRNA. In turn, control cells, which were obtained by transduction of A549 cells with the empty vector, were termed A549.CTRL. The results demonstrated that A549.shRNA cells were characterized by a statistically significant decreased level of *POSTN* mRNA and highly decreased binding of anti-POSTN antibodies to cell lysates compared to control cells (A549.CTRL) (*** *p* < 0.001); (Figure 8A,C). These results were also confirmed by immunofluorescence analysis. We noticed significantly decreased fluorescence intensity in A549.shRNA cells compared to control cells (*** *p* < 0.001) (Figure 8B).

### 2.6. Effects of POSTN Silencing on A549 Cell Migration and Invasion In Vitro

**Figure 9:**
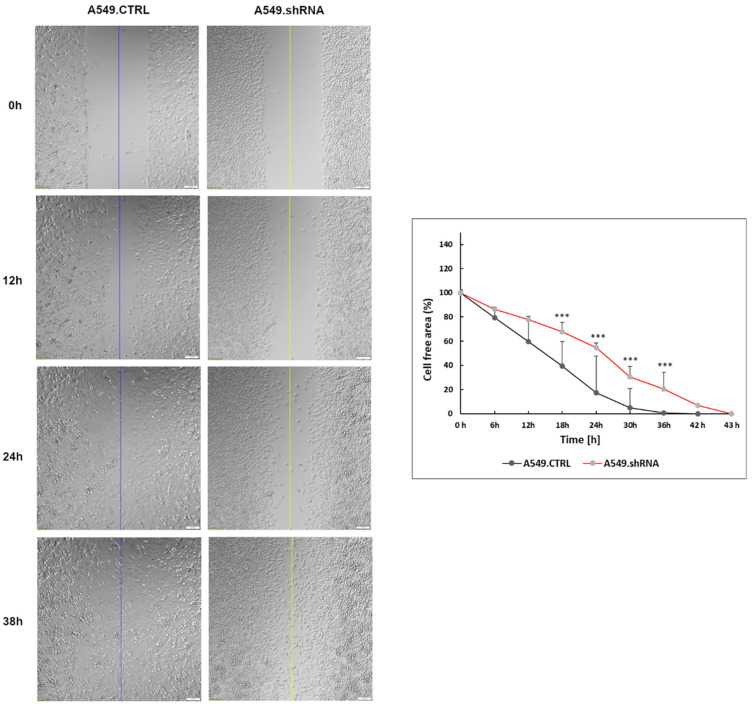
The effect of periostin (POSTN) silencing on the migration of lung cancer cells. Compared to control cells (A549.CTRL), migration of A549.shRNA cells was identified by wound-healing assays. Images were obtained each hour up to 43 h following the injury, always from the same place (3 pictures per well), and analyzed using CellSense software (Olympus, Hamburg, Germany, RRID: SCR_016238). The cell-free area (%) was calculated. Data were analyzed using Bonferroni multiple comparison tests (*** *p* < 0.001). Magnification 10×. Bar = 100 µm.

The wound-healing assay was used to evaluate the effects of POSTN silencing on the migration of A549 cells. The results of the wound healing assay indicated that A549.shRNA cells migrated significantly slowly to close the scratched wounds compared to control cells transduced only with an empty vector (A549.CTRL). There was a statistically significant difference in migration distance in the control and A549.shRNA cells at 18, 24, 30, and 36 time periods, respectively (*** *p* < 0.001; Bonferroni multiple comparisons) (Figure 9).

**Figure 10:**
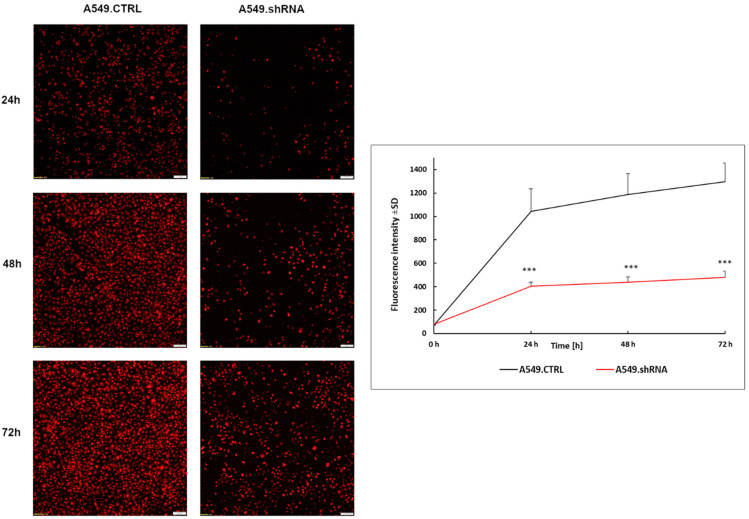
Silencing of periostin (POSTN) affects the invasiveness of lung cancer cell lines. Reduced invasion through the Matrigel matrix was observed in fluorescently labeled A549.shRNA cells compared to A549.CTRL cells. Data are presented as mean ± SD. Bonferroni multiple comparison test, *** *p* < 0.001. Magnification 10×. Bar = 100 µm.

Furthermore, a Transwell invasion assay was conducted to evaluate the effects of POSTN silencing on the invasive abilities of A549 cells. The results showed that the ability of lung cancer cells to invade through the Matrigel™ matrix was significantly decreased in the fluorescently labeled A549.shRNA cells in comparison with labeled A549.CTRL cells (Figure 10). A statistically significant decrease in invasiveness was found in lung cancer cells, silencing POSTN (A549.shRNA) compared to the control cells (A549.CTRL) at the 24th, 48th, and 72nd hours of the experiment (*** *p* < 0.001, respectively; Bonferroni multiple comparisons) (Figure 10). Taken together, these results suggested that POSTN silencing could inhibit the migratory and invasive abilities of A549 cells in vitro.

### 2.7. POSTN Silencing Decreases the Expression of Integrin-Signaling Pathway-Related Protein

**Figure 11:**
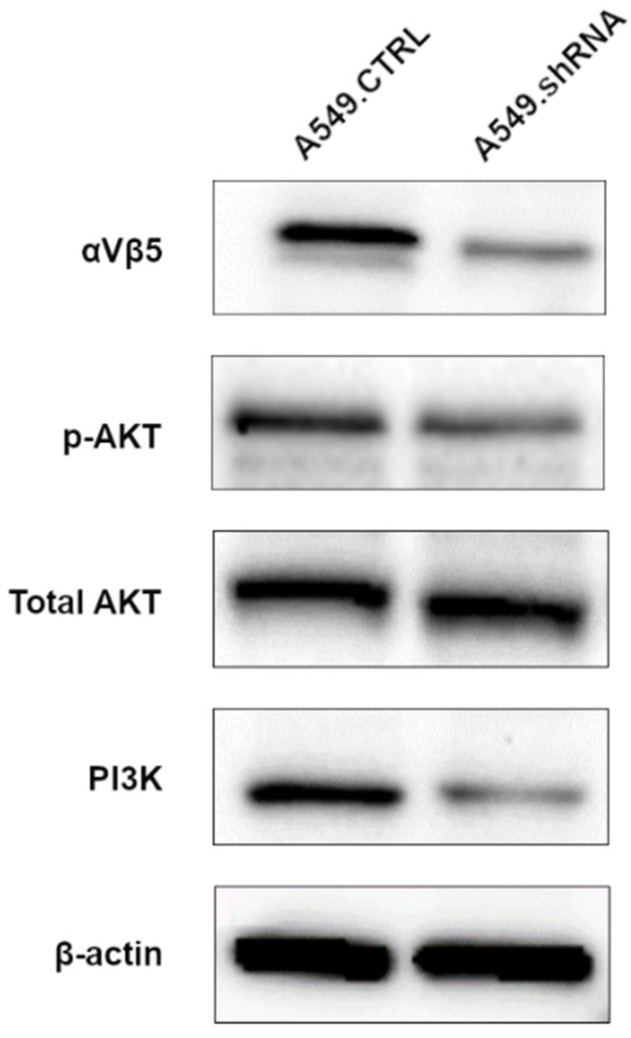
Effects of periostin (POSTN) silencing on integrin-signaling-pathway-related protein. Western blotting assay was performed to detect integrin-αvβ3, p-AKT, total AKT, and PI3K in A549.shRNA cells compared to A549.CTRL cells. β-actin protein was used as loading controls.

In order to assess the underlying mechanism of the POSTN-mediating integrin-signaling pathway, Western blotting analysis was performed to detect the expression of integrin-αvβ3, AKT, pAKT, and PI3K. As shown in Figure 11, we noticed a decreased protein expression level of integrin-αvβ3 and PI3K in the cells representing the loss-of-function phenotype (A549.shRNA) compared to A549.CTRL cells. Furthermore, Western blot analysis showed a decreased level of AKT phosphorylation in A549.shRNA cells, while the total amount of AKT remained unchanged. The results suggest that silencing of POSTN might reduce the expression of integrin-signaling-pathway-related proteins in order to inhibit NSCLC cell invasion and metastasis.

### 2.8. POSTN Silencing Downregulates the Protein Expression Levels of MMP-2 and MMP-2 Activity

**Figure 12:**
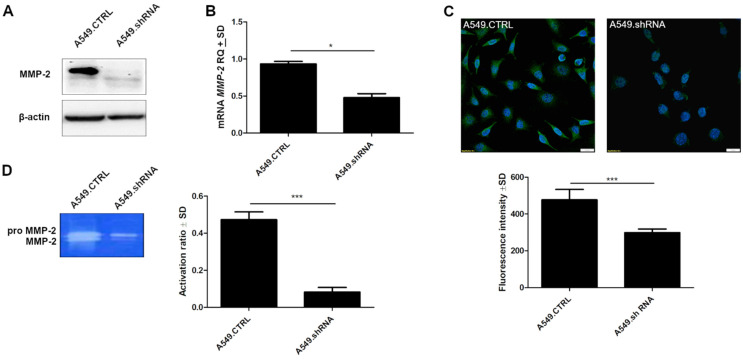
Effects of periostin (POSTN) silencing on MMP-2 expression and MMP-2 activity. (**A**) MMP-2 protein was detected by Western blot analysis. β-actin served as an internal control. Western blotting shows marked changes in the expression level of MMP-2 in control (A549.CTRL) cells and A549.shRNA cells transfected with POSTN-specific short hairpin. (**B**) Relative quantification (RQ) of mRNA expression levels of *MMP-2* mRNA in control cells (A459.CTRL) and A549.shRNA cells (* *p* < 0.05). (**C**) Comparison of MMP-2 expression levels detected by confocal microscopy in control A549.CTRL cells and in the cells representing the loss-of-function phenotype (A549.shRNA) (*** *p* < 0.001). Objective 60×/1.40 Oil; pinhole airy 1.25. Bar = 20 µm. (**D**) MMP-2 activity was analysed using gelatin zymography (*** *p* < 0.001). RQ, relative quantification.

To explain the effect of POSTN on the regulation of intracellular molecules involved in aggressive cell behavior, we examined its impact on MMP-2 expression. Western blot analysis indicated that the protein expression level of MMP-2 was markedly decreased in A549.shRNA cells compared to A549.CTRL (Figure 12A). These results were confirmed by molecular studies of lung cancer cell lines. We noticed significantly decreased expression of *MMP-2* mRNA in the cells representing the loss-of-function phenotype (A549.shRNA) compared to control cells (A549.CTRL) (* *p* < 0.05) (Figure 12B). Furthermore, we showed significant differences in fluorescence intensity between A549.shRNA cells transfected with POSTN-specific short hairpin and control A549.CTRL cells (*** *p* < 0.001) (Figure 12C).

Moreover, we detected the effect of POSTN silencing on MMP activity, which was correlated with basement membrane degradation and cancer cell invasion. The activity of MMP-2 was measured by zymography (Figure 12D). The results of zymography assays showed that in A549.shRNA cells, the activity ratio of MMP-2 was significantly decreased compared to control cells (A549.CTRL) transduced only with an empty vector (*** *p* < 0.001) (Figure 12D). This result suggested that enhanced activity of MMP -2 may contribute to POSTN-induced NSCLC invasiveness.

### 2.9. Effects of Increased POSTN on MMP-2 Expression

**Figure 13:**
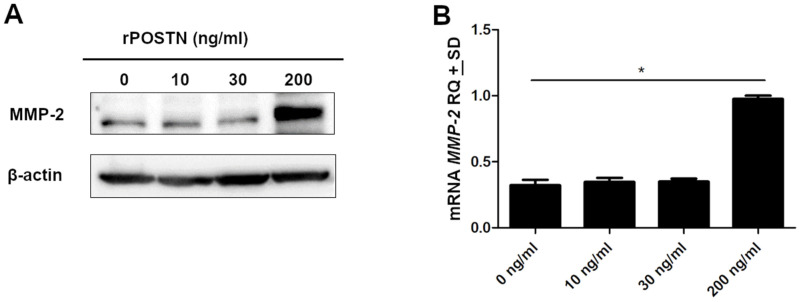
Effects of increased periostin (POSTN) on MMP-2 expression. A549 cells were cultured in the serum-free medium for 12 h, followed by treatment with various concentrations of recombinant POSTN (rPOSTN) for a further 12 h. (**A**) MMP-2 expression was determined by Western blot analysis. β-actin served as an internal loading control. (**B**) *MMP-2* mRNA expression level was analyzed by real-time PCR (* *p* < 0.05). RQ, relative quantification.

To evaluate the effects of increased POSTN on MMP-2 expression, A549 cells were stimulated with various concentrations of recombinant protein-POSTN. The level of MMP-2 expression was not significantly changed by the addition of 10 or 30 ng/mL POSTN but markedly increased by the application of 200 ng/mL POSTN (Figure 13A). Furthermore, our results demonstrated that the expression level of mRNA *MMP-2* was statistically significantly higher at a concentration of 200 ng/mL recombinant POSTN compared to those in 0 ng/mL (* *p* < 0.05) (Figure 13B).

## 3. Discussion

POSTN is an extracellular matrix N-glycoprotein that is a major constituent of the desmoplastic stroma around solid tumors [18]. POSTN can directly exert its functions on tumor cells in paracrine or autocrine mode. Additionally, POSTN supports oncogenesis not only by activating intracellular pathways but also through its impact on ECM desmoplasia. The desmoplastic stroma of a malignant neoplasm constitutes a tumor microenvironment that supports tumor growth and invasion. Nevertheless, specific molecular mechanisms defining how POSTN remodels distinct tumor microenvironments have not been fully accounted for [18,25].

POSTN upregulation has been demonstrated for many cancer types, such as non-small cell lung cancer (NSCLC) [24,26], invasive ductal breast cancer (IDC) [27], pancreatic [28], and ovarian cancer [29], and is consequently defined as a tumor-enhancing factor [17]. Only a few reports in bladder cancer and osteosarcoma have shown POSTN as a tumor-inhibiting factor [21,30]. To confirm this, analysis of POSTN expression is required in a larger cohort of various cancer cases.

Consistent with studies related to other cancers [18,31], our results indicated that POSTN was involved in the motility and in vitro invasive potential of lung cancer cells. The data suggest that POSTN plays a crucial role in the multistep cascade process of cancer metastasis. Invasion of the basement membrane and ECM is critical for metastasis of NSCLC, which depends on degradation of these components, particularly by MMP, proteolytic enzymes widely associated with increasing cancer-cell growth, tumor invasion, and metastasis [7,32,33,34].

Our study is the first in which the epithelial cytoplasmic expression of POSTN was studied on a large population of patients with NSCLC as well as in two histological subtypes (AC and SCC) with regard to patients’ clinicopathological factors. Our results confirmed the increased expression of POSTN in cancer cells of NSCLC compared to NMLT, suggesting that POSTN could be related to the process of carcinogenesis in NSCLC. The results of the present study are in line with our previous observations regarding POSTN expression in cancers cells of invasive ductal carcinoma (IDC) [35] as well as in the stromal compartment (CAFs) of NSCLC [24]. Moreover, LCM, which was used to determine the *POSTN* mRNA expression levels in NSCLC cancer cells, confirmed the TMA IHC observations. We noticed a significantly higher *POSTN* mRNA expression in NSCLC cancer cells compared to NMLT. In addition, in our study, we showed a positive correlation of POSTN expression in cancer cells with MMP-2 expression level in NSCLC cells, which is in line with our results of in vitro studies indicating that POSTN could regulate lung cancer cell invasiveness by modulating the expression and activity level of MMP-2. Furthermore, we noticed, for the first time, a strong positive significant correlation of POSTN in cancer epithelial cells with stromal POSTN expression (CAFs) in the whole cohort of patients as well as in the particular histological types (i.e., AC and SCC). This relationship indicates the potential interaction between cancer cells and stromal cells of NSCLC. It has been confirmed that a direct interaction between cancer-associated fibroblasts (CAFs) and cancer cells as well as the cross-talk between cells and the ECM can result in further changes in both cell types, and hence a more efficient CAF-led cancer cell invasion [11]. Cancer cells can use the ECM proteins secreted by their neighboring stromal cells, and they themselves form a supportive microenvironment for the initiation and growth of a primary tumor and metastasis [36]. Several studies suggested that POSTN could be involved in facilitating the interaction between cancer cells and the tumor microenvironment to promote cell migration. Such interactions are mostly mediated by interactions with receptors of the integrin family. Some studies indicated that POSTN-integrin interaction could inhibit the ECM–integrin interaction and trigger both the intracellular signaling and activation of some genes connected with tumor progression [37]. It has been recently shown that POSTN may support adhesion and migration of ovarian epithelial cancer cells by interacting with αvβ3 and αvβ5 integrins [11,29]. Orecchia et al. [38] also showed that the proliferating activity of melanoma cells was inhibited by the addition of antibodies directed against POSTN elements involved in the interaction with both αvβ3 and αvβ5 integrins, which showed that such an interaction was crucial for tumor growth [11]. Therefore, it is believed that the identification of molecules that mediate the association of cancer cells with CAFs is the most crucial challenge. It is also significant to identify signaling pathways and molecules in cancer cells that can be activated upon direct interaction with CAFs. Therefore, the analysis of interactions between these cells will be the subject of our future research.

Moreover, in this study, an analysis of expression intensities of POSTN concerning clinicopathological parameters showed a significant increase in POSTN with increasing clinical stages (TNM). Additionally, we noticed an increased level of epithelial POSTN with increasing tumor size (pT) and lymph node metastases, both in the whole cohort and in the particular histological subtypes (AC and SCC). These findings are in accordance with the observations of Soltermann et al. [39], who showed that in NSCLC, epithelial POSTN was also significantly associated with several clinicopathological parameters such as squamous cell carcinoma histotype, higher stage, and higher pT as well as larger tumor size. However, it should be emphasized that compared to our studies, those authors used a different patient pool size. A similar study was obtained by Zhu et al. [40], who indicated that high POSTN levels in cancer cells of ovarian cancers were correlated with advanced late stages (III/IV) and cancer recurrence. The above observations were also confirmed in the case of renal cell carcinoma by Morra et al. [41]. In their study, they showed that higher levels of POSTN in cancer epithelial cells correlated with higher tumor stage, lymph node metastases, and poor overall survival. Interestingly, in line with our previous studies [24,35], we also showed that the intensity of immunoreactivity of POSTN in epithelial cancer cells increased with the malignancy grade of the tumors and had an impact on patient overall survival. The survival analysis demonstrated that a high epithelial expression of POSTN in NSCLC and in AC and SCC subtypes was associated with poor patient outcomes. This indicates the influence of POSTN in cancer development. Moreover, a multivariate analysis showed that POSTN expression in epithelial cancer cells can be an independent positive prognostic factor in the whole NSCLC patient cohort as well as in AC and SCC groups. Similarly, Ben et al. [42] demonstrated that in pancreatic ductal adenocarcinoma (PDAC), high POSTN expression in cancer epithelial cells was indicative of poor prognosis compared to the adjacent tissue. Furthermore, studies by Riener et al. [43], related to liver tumors, indicated that POSTN expression in cancer epithelial cells was associated with reduced overall survival and correlated with tumor grade. A similar trend was found in our study. Similar results were also reported in oesophageal squamous cell carcinoma by Wang et al. [44], who found that high POSTN expression correlated with poor prognosis and shorter overall survival, which is in line with the tendency demonstrated in our study.

These experimental results, obtained using clinical material, were further supported by in vitro studies. Our results indicated that POSTN silencing using shRNA (short-hairpin RNA) significantly inhibited the migratory and invasive capabilities of lung cancer cells (A549.shRNA) compared to control A549.CTRL cells. We conducted additional experiments to consolidate the mechanistic study of the effect of POSTN on migration and invasion. Our study findings indicated that protein expressions of integrin-αvβ3 and PI3K/pAKT were reduced in the cells representing the loss-of-function phenotype (A549.shRNA) compared to control cells, indicating the silencing of POSTN in the integrin-signaling pathway during the course of NSCLC. POSTN is capable of binding to integrins, including -αvβ3, -αvβ5, and -α6β4, thereby promoting activation of specific integrin-mediated signaling pathways such as Akt/PI3K signaling pathways, which leads to increased cell survival, angiogenesis, invasion, and metastasis. Consequently, we may assume that POSTN silencing may inhibit NSCLC progression by blocking the αvβ3 integrin/ PI3K/AKT signaling pathway. Interestingly, in the present study, we also found that POSTN silencing statistically significantly decreased the protein expression level of MMP-2 as well as the enzyme activity of MMP-2 in A549.shRNA cells, which likely contributes to decreasing the migratory and invasive ability of lung cancer cells. Thus, the results of our research indicate that POSTN silencing might modulate the tumor microenvironment by affecting MMP-2, a protein acting as a metastasis-associated factor of the tumor microenvironment, which serves an important role in the degradation of the basement membrane and the invasion of cancer cells [6,7,8]. Therefore, our results suggest that POSTN promotes the invasive ability of lung cancer cells, at least partly via tumor microenvironment factor MMP-2, highlighting MMP-2 as an effector of POSTN signaling in lung cancer cells [45]. Furthermore, to the best of our knowledge, this is the first study to show a possible relationship between the knockdown of POSTN expression in lung cancer cells in vitro and the downregulation of MMP-2. The αvβ integrin/ERK signaling pathway is one of the mechanisms by which MMP-2 expression is upregulated in tumor cells [46]. As a result, POSTN could also induce the upregulation of MMP-2 expression via the αvβ3 integrin/ERK pathway. Such a mechanism was demonstrated by Watanabe et al. [47]. The functional analyses in human periodontal ligament cells revealed that POSTN regulated MMP-2 expression via the αvβ3 integrin/ERK signaling pathway. Moreover, Yan et al. [48] found that POSTN overexpressing 293T cells showed increased MMP-9 activation. In turn, overexpression of POSTN in the bronchial epithelial cell line BEAS-2B promoted the epithelial expression of MMP-2 and MMP-9 in a TGF-β-dependent manner [49]. Similar results were also reported in glioma by Wang et al. [45]. In their study, they showed that POSTN promoted glioma cell invasiveness in vitro, accompanied by MMP-9 expression. A recent study of renal cell carcinoma cells [33] also demonstrated that POSTN overexpression increased the activity of MMP-2 and MMP-9. Furthermore, they noticed that FAK knockdown attenuated MMP levels, cell migration, and invasion, which were all enhanced by POSTN, suggesting that POSTN plays a critical role in the multistep cascade process of cancer metastasis [33]. Similar conclusions were drawn by Ouanouki et al. [50], who indicated that silencing of POSTN inhibited U-87 glioblastoma cell migration and invasive potential, which is in line with the tendency demonstrated in our study.

In view of the above facts, we also examined the impact of the recombinant POSTN protein on the expression of MMP-2 in the A549 lung cancer cell line. It was found that recombinant POSTN enhanced MMP-2 expression in a concentration-dependant manner, which confirms the results obtained earlier on A549 lung cancer cells transfected with POSTN-specific short hairpin (A549.shRNA). We thus suggest that POSTN increases the invasive ability of lung cancer cells by increasing their migratory properties, thus affecting the expression of matrix metalloproteinases such as MMP-2, an endopeptidase playing a crucial role in the carcinogenesis of lung cancer, with functions in cell proliferation, tumor invasion, and metastasis. Our findings, indicating that exogenous POSTN increased MMP-2 expression, correspond to previous data from Burgess et al. [49], who reported that POSTN induced the expression of MMP-9 in differentiated primary epithelial cells. Similarly, Kanno et al. [22] revealed that a high concentration of recombinant POSTN promoted cell migration in pancreatic cancer cells, which is also in line with the tendency observed in our studies.

In summary, the results of this study demonstrate the important functional and molecular mechanisms of POSTN in tumor invasion. Silencing of POSTN could inhibit the migration and in vitro invasive potential of lung cancer cells, most probably via the downregulation of MMP-2 expression and activity as well as integrin-signaling related proteins. Furthermore, our data indicated that epithelial POSTN expression could be an independent negative prognostic factor in NSCLC and could represent a potential future therapeutic target.

## 4. Materials and Methods

### 4.1. Patient Cohort

In total, 715 NSCLC and 110 adjacent NMLT samples were collected from patients treated in the Department of Thoracic Surgery of Wroclaw Medical University between 2007 and 2017. The paraffin-embedded specimens included 110 NMLT, 298 AC, 370 SCC, and 47 large cell carcinomas (LCC). The histological tumor type was evaluated based on the World Health Organization Classification [51] by two independent pathologists and was confirmed by immunohistochemical staining for the marker proteins TTF-1 (AC marker) and p63 (LSCC marker). The pTNM classification was made in accordance with the recommendations of the International Association for the Study of Lung Cancer (IASLC) [52].

The experiment was conducted with the ethical standards and the approval of the Bioethics Committee of the Wroclaw Medical University.

Laser microdissection was performed on 10 frozen NSCLC fragments and 6 NMLTs as the control. Clinicopathological patient characteristics are given in Table 2.

### 4.2. Construction of Tissue Microarray (TMA) and Immunohistochemistry (IHC)

TMAs were constructed as described previously [24]. Briefly, three morphologically representative tumor cores from the center of the tumor, with a 1.5 mm core size, were assembled into the TMA using the TMA Grand Master (3DHistech) automatic tissue microarrayer.

To investigate POSTN, MMP-2, TTF-1, and p63 protein expression in TMAs were analyzed by IHC, as described before [24]. POSTN was detected using a primary rabbit anti-human polyclonal antibody (NBP1-82472; Novus Biologicals) diluted 1:200, incubated for 20 min at RT. MMP-2 was detected using rabbit anti-human MMP-2 antibody (10373-2-AP Proteintech, Manchester, UK) diluted at 1:200, incubated for 20 min at RT. Furthermore, in order to detect TTF-1 and p63 expression, specific primary antibodies were used: anti-TTF-1(mouse, Dako, Cat# IR056, RRID:AB_2755006; dilution: 1:50, 20 min RT) and anti-p63 (1:300 dilution; Dako, Glostrup, Denmark/Santa Clara, CA, USA, Cat# IR622, RRID:AB_2755007, 20 min RT). IHC reactions were performed using Dako Autostainer Link48 (Dako, Glostrup, Denmark). The visualization of the reactions was carried out using EnVision™ FLEX High pH (Link) reagents (Dako), in accordance with the manufacturer’s protocols.

Expressions of POSTN and MMP-2 were evaluated using the immunoreactive score (IRS) scale by Remmele and Stegner [53], as described before [24,27], with methodological standardization to ensure result reproducibility. Two independent pathologists evaluated all specimens using an OLYMPUS BX-41 light microscope (Olympus, Hamburg, Germany).

### 4.3. Laser Capture Microdissection (LCM)

LCM was performed, essentially as described previously [24], using the MMI CellCut Plus system (MMI). Furthermore, total RNA was extracted from the microdissection samples by using the RNeasy Micro Kit (Qiagen, Hilden, Germany) according to the company’s protocols.

### 4.4. Reverse Transcription–Quantitative PCR (RT–qPCR)

Total RNA was isolated from the cell lines using RNeasy Mini Kit (Qiagen, Hilden, Germany); for the reactions, the following sets of primers and TaqMan probes were used: Hs00170815_m1 for *POSTN*, Hs01548727_m1 for *MMP-2* and Hs99999903_m1 for *ACTB* (Applied Biosystems; Thermo Fisher Scientific, Inc., Carlsbad, CA, USA); the relative expressions of *POSTN* and *MMP-2* mRNA (RQ) were calculated with the ΔΔCt method [54], as previously described [24,35].

### 4.5. Cell Lines

A549 (ATCC, Cat# CCL-185), NCI-H1703 (ATCC, Manassas, VA, USA; Cat# CRL-5889), and NCI-H522 (ATCC, Cat# CRL-5810) cell lines were obtained from the American Type Culture Collection (ATCC). Both NCI-H1703 and NCI-H522 cell lines were cultured in RPMI-1640 medium (Lonza, Basel, Switzerland) supplemented with 10% fetal bovine serum (FBS) (Sigma-Aldrich, Saint Louis, MO, USA) and 2 mM L-glutamine. In turn, A549 cells were routinely cultured in F12K supplemented (to final concentration) with 10% FBS, and 2 mM L-glutamine (Lonza, Basel, Switzerland).

LentiX 293T cells were purchased from Clontech Laboratories (Terra Bella Avenue, Mountain View, CA, USA) and cultured in αMEM supplemented with 10% FCS (Invitrogen), 2 mM L-glutamine, 100 U/mL streptomycins, and 0.1 mg/mL penicillin (complete αMEM). The conditions of cell culture were as follows: temperature 37 °C and 5% CO_2_ concentration.

### 4.6. Virus Production, Transductions and Cell Maintenance

For lentivirus production packaging LentiX 293T cells were co-transfected at 50–60% confluence with 20 μg of POSTN MISSION shRNA (TRCN0000123055) expression vector or SHC016 PLKO.1-PURO NON-TARGET (SHC016-1EA) control vector (MISSION® shRNA Plasmid DNA system, Sigma-Aldrich, Saint Louis, MO, USA), 10 μg pMDL-g/p-RRE, 5 μg pRSV-REV, and 5 μg pMk-VSVG (Addgene, Watertown, MA, USA) using polyethyleneimine (Sigma-Aldrich, Saint Louis, MO, USA) at a concentration of 1 mg/mL. Culture supernatants containing virus particles were collected 48 h after transfection and clarified through a 0.45 μm pore size filter (Millipore, Billerica, MA, USA).

The virus-containing supernatant was concentrated 100× on an Amicon Ultra-15K:100.000 (Millipore). A549 cells (2 × 10^4^) were transduced with the concentrated virus stock by centrifuging (2460× *g*) at 23 °C for 2 h. The cells were selected for puromycin resistance (1 µg/mL) for 1 week and maintained in a medium containing 1 µg/mL puromycin.

### 4.7. Protein Isolation, SDS-PAGE, and Western Blotting

Protein preparation and Western blotting were performed as described previously [24]. The following antibodies were used: primary rabbit anti-human POSTN polyclonal antibody (NBP1-82472; Novus Biologicals, Littleton, CO, USA) diluted at 1:500, rabbit anti-human MMP-2 antibody (10373-2-AP Proteintech, Manchester, UK) diluted at 1:1000, rabbit anti-human α_V_β_3_ antibody (NBP2-67557; Novus Biologicals) diluted at 1:1000, rabbit anti-human pAKT antibody (#4058 Cell Signaling Technology, Danvers, MA, USA), diluted at 1:1000, rabbit anti-human AKT antibody (#9272 Cell Signaling Technology) diluted at 1:1000, and mouse anti-human PI3K antibody (MA5-17149, Thermo Fisher Scientific, Carlsbad, CA, USA) diluted at 1:1000. The results were documented in a Chemi-Doc XRS Molecular Imager apparatus (Bio-Rad, RRID: SCR_014210). β-actin (Abcam ab8229, Cambridge, UK) was used as a loading control.

### 4.8. Wound-Healing Assay

We used the ibidi Culture-Insert in µ-Dish^35mm, high^ system (ibidi GmbH, Munich, Germany) with two chambers to separate different cell types in order to measure the migratory properties of A549.shRNA and A549.CTRL cells. For the wound-healing assay, cells were serum-starved for 24 h for cell synchronization. Then, 5.0 × 10^5^ cells were seeded into each chamber and grown to confluence in F12K medium containing 2% FBS for 24 h. After incubation for an additional 48 h, cell migration was analyzed in six different microscopic fields and calculated as the percentage of wound healing. Images of the initial wound and the movement of cells into the scratched area were captured using a Fluoview FV3000 confocal laser scanning microscope (Olympus, Hamburg, Germany, RRID: SCR_017015) coupled with CellSens software version 3.2 (Olympus, RRID: SCR_016238).

### 4.9. Transwell Invasion Assay

Invasion assays were performed using a BioCoat MatrigelTM invasion chamber (pore size of 8 μm) and an HTS FluoroBlok membrane insert (pore size of 8 μm) for a 24-well plate (Corning, MA, USA), respectively. For the invasion assay, cells were firstly starved using serum-free media for 24 h. A549.shRNA and A549.CTRL cells were collected by trypsinization, resuspended in 1 mL PBS at a density of 10^6^/mL, and labeled with CellTracker™Red CMTPX (Invitrogen, Carlsbad, CA, USA) at 37 °C for 30 min. For the invasion assay, 200 µL of warm (37 °C) culture medium (F12K) was added to the interior of the insert wells and left for 2 h at 37 °C, 5% CO2 to rehydrate. After rehydration, 5 × 10^4^/200µL of cell suspension was applied to the apical chambers. The lower chamber was filled with 600 µL of F12K containing 10% FBS as a chemoattractant. The invasion assay was conducted for 24, 48, and 72 h. The fluorescence of the invaded cells was read at wavelengths of 577 and 602 nm (for excitation and emission, respectively) on a bottom reading Infinite M200 Pro fluorescent microplate reader (Tecan, Männedorf, Switzerland). Furthermore, the invaded cells on the underside were viewed under a Fluoview FV3000 confocal laser scanning microscope (Olympus, RRID: SCR_017015). The same parameters were used to capture the microscopic images. Wells without cells were a blank to subtract for background.

### 4.10. Immunofluorescence (IF)

The methodology for the preparation of the IF was performed as described previously [35]. The cells were incubated overnight at 4 °C with primary anti-POSTN antibodies (NBP1-82472; Novus Biologicals, Littleton, CO, USA) and with an anti-MMP-2 antibody (10373-2-AP Proteintech, Manchester, UK). Subsequently, secondary Alexa Fluor 568-conjugated donkey anti-rabbit antibodies (1:2000, 1h/RT; Abcam, Cambridge, UK, Cat# ab175470, RRID: AB_2783823) and Alexa Fluor 488-conjugated goat anti-rabbit antibodies were applied (1:2000, 1h/RT; Abcam, Cat# ab150089, RRID:AB_2755130). The slides were analyzed using a Fluoview FV3000 confocal laser scanning microscope (Olympus, RRID: SCR_017015) coupled with CellSense software (Olympus, RRID: SCR_016238).

### 4.11. Gelatin Zymography

The gelatinolytic activity of MMP-2 was measured by gelatin zymography as described previously [55]. The clear band against a blue background representing the activity of MMP-2 was measured using a gel image system (ImageMaster 1D analysis software version 2.01, Nonlinear Dynamics, Newcastle, UK). The results for each sample were expressed as the mean ratio of the percentages of active to inactive forms (the activation ratio).

### 4.12. Statistical Analysis

The results were analyzed using Prism 5.0 software (GraphPad Software, San Diego, CA, USA) and Statistica 13.3 (Tibco Software Inc., Palo Alto, CA, USA). To compare the differences in the expression of the markers in all groups of patients and the clinicopathological data, the following were used: Student’s *t*-test, unpaired *t*-tests, Mann–Whitney test, Kruskal–Wallis test with Dunn’s multiple comparison test, and Bonferroni post hoc multiple comparison test. The correlation analysis was conducted using Spearman’s rank correlation coefficient. Survival analysis was performed using the Kaplan–Meier method. The Gehan–Breslow–Wilcoxon method and the univariate and multivariate Cox analyses of survival were performed to assess the analysis of survival. The results were considered statistically significant when *p* < 0.05.

## 5. Conclusions

Our study has indicated that the expression of POSTN in cancer cells may be an independent prognostic factor for survival in patients with NSCLC and the particular histological subtypes, i.e., AC and SCC. Furthermore, we noticed a significant correlation of POSTN expression in cancer cells with MMP-2 expression levels in NSCLC. These experimental results were further supported by in vitro studies. To clarify the role of POSTN in lung cancer progression, we analyzed the effect of POSTN silencing on the migration and invasiveness of lung cancer A549 cells. It was found that lung cancer cells with the silenced expression of POSTN (A549.shRNA) were characterized by decreased migratory capacity and invasiveness in vitro compared to control cells. We showed that the silencing of POSTN might reduce the expression of integrin-signaling-pathway-related proteins in order to inhibit NSCLC cell invasion and metastasis.

**Figure 14:**
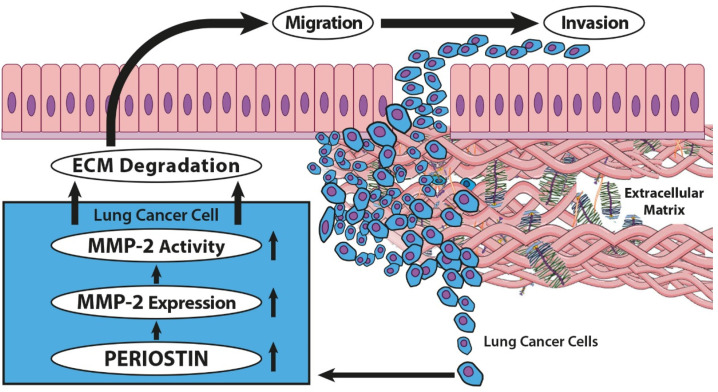
Schema of the signaling pathway involved in the invasion of lung cancer. POSTN might regulate lung cancer cell invasiveness by modulating the expression and activity level of MMP-2, a protein acting as a metastasis-associated factor of the tumor microenvironment, which serves an important role in the degradation of the basement membrane and the invasion of cancer cells. The figure is based on Cui et al. [56], modified.

Interestingly, our results showed for the first time that POSTN might regulate lung cancer cell invasiveness by modulating the expression and activity level of MMP-2, an enzyme that degrades the basement membrane and components of the ECM, allowing for the invasion of tumor cells and proliferation in the metastatic environment (Figure 14). These findings may indicate the potential role of POSTN in the pathogenesis and progression of NSCLC.

## Figures and Tables

**Table 1:** Univariate and multivariate Cox analyses of overall survival of patients with (**A**) non-small cell lung cancer (NSCLC) as well as (**B**) squamous cell carcinoma (SCC) and adenocarcinoma (AC) of the lung.

(**A**)								
**Overall Survival (OS)**								
**Clinicopathological Parameters**				**NSCLC**				
				**Univariate Analysis**			**Multivariate Analysis**	
				***p*** **-Value**	**HR (95% HR CI)**		***p*** **-Value**	**HR (95% HR CI)**
Age (<62 vs. >62)				**0.0099**	1.2915 (1.0635–1.5684)		**0.0011**	1.3867 (1.1389–1.6885)
POSTN cancer cells (low vs. high)				**<0.0001**	1.8496 (1.5203–2.2502)		**<0.0001**	1.5401 (1.2595–1.8831)
Ki-67 (median)				0.5862	0.9456 (0.7729–1.1567)		-	-
TTF-1				0.7634	0.9908 (0.9333–1.052)		-	-
p63				0.0938	0.9517 (0.898–1.0084)		-	-
Smoking history				0.2842	1.01 (0.9918–1.0286)		-	-
Stage (I–II vs. III–IV)				**<0.0001**	2.1399 (1.7488–2.6185)		**0.1108**	1.2661 (0.9474–1.692)
Grade (G1 vs. G2-G3)				0.7886	1.0539 (0.718–1.5471)		-	-
Tumour size (T1-T2 vs. T3-T4)				**<0.0001**	1.9758 (1.6174–2.4136)		**0.0001**	1.6143 (1.2715–2.0495)
Lymph nodes involvement (N0 vs. N+)				**<0.0001**	1.8288 (1.5009–2.2284)		**0.0004**	1.5767 (1.223–2.0327)
pM				0.6951	1.3087 (0.3408–5.0262)		-	-
(**B**)								
**Overall Survival (OS)**								
**Clinicopathological Parameters**	**SCC**				**AC**			
	**Univariate analysis**		**Multivariate analysis**		**Univariate analysis**		**Multivariate analysis**	
	***p*** **-Value**	**HR (95% HR CI)**	***p*** **-Value**	**HR (95% HR CI)**	***p*** **-Value**	**HR (95% HR CI)**	***p*** **-Value**	**HR (95% HR CI)**
Age (<62 vs. >62)	**0.0463**	1.3453 (1.0049–1.801)	**0.0279**	1.3912 (1.0365–1.8673)	0.1792	1.2257 (0.9108–1.6493)	-	-
POSTN cancer cells (low vs. high)	**0.0001**	1.827 (1.3593–2.4556)	**0.0045**	1.5597 (1.1476–2.1196)	**<0.0001**	2.0868 (1.548–2.8132)	**0.0025**	1.6236 (1.1858–2.223)
Ki-67 (median)	0.5593	0.915 (0.679–1.233)	-	-	0.7894	0.9599 (0.7109–1.2962)	-	-
TTF-1	0.2344	1.0831 (0.9496–1.2353)	-	-	**0.0469**	0.8994 (0.8102–0.9986)	0.2284	0.9345 (0.8369–1.0434)
p63	0.0675	0.8935 (0.792–1.0081)	-	-	0.7377	0.978 (0.8589–1.1138)	-	-
Smoking history	0.3124	1.01 (0.9907–1.0298)	-	-	0.0662	1.4783 (0.9742–2.2431)	-	-
Stage (I vs. II–IV)	**0.0001**	1.9025 (1.394–2.5965)	0.3043	1.2635 (0.8087–1.9741)	**<0.0001**	2.3709 (1.751–3.2103)	0.3528	1.2374 (0.7896–1.9392)
Grade (G1 vs. G2-G3)	0.2417	0.6367 (0.299–1.3557)	-	-	0.2646	1.3118 (0.8144–2.113)	-	-
Tumour size (T1-T2 vs. T3-T4)	**<0.0001**	1.8701 (1.3894–2.517)	**0.0221**	1.5217 (1.0623–2.1798)	**<0.0001**	2.0099 (1.4645–2.7583)	**0.0362**	1.4849 (1.0258–2.1496)
Lymph nodes involvement (N0 vs. N+)	**0.0039**	1.5464 (1.1504–2.0788)	0.1079	1.3633 (0.9343–1.9892)	**<0.0001**	2.3189 (1.7176–3.1306)	**0.0026**	1.8544 (1.2414–2.7701)
pM	-	-	-	-	0.7036	1.3302 (0.3058–5.7855)	-	-

Significant *p*-values are given in bold. HR—hazard ratio; CI—confidence interval; POSTN—periostin; NSCLC—non-small cell lung cancer; AC—adenocarcinoma, SCC—squamous cell carcinoma. Values in bold are statistically significant.

**Table 2:** Clinical and pathological characteristics of patients with non-small cell lung carcinoma (NSCLC).

Characteristics	NSCLC		AC		SCC		LCC	
	*N* = 715	%	*N* = 298	%	*N* = 370	%	*N* = 47	%
Age								
≤62	36	5.03%	21	7.05%	10	2.70%	26	55.32%
>62	679	94.97%	277	92.95%	360	97.30%	21	44.68%
Tumor size								
pT1	184	25.73%	74	24.83%	93	25.14%	14	29.79%
pT2	313	43.78%	145	48.66%	155	41.89%	18	38.30%
pT3	149	20.84%	52	17.45%	85	22.97%	9	19.15%
pT4	69	9.65%	27	9.06%	37	10.00%	6	12.77%
Tumor grade								
G1	52	7.27%	37	12.42%	12	3.24%	2	4.26%
G2	565	79.02%	205	68.79%	301	81.35%	39	82.98%
G3	98	13.71%	56	18.79%	57	15.41%	6	12.77%
Lymph node involvement								
pN0	471	65.87%	193	64.77%	240	64.86%	34	72.34%
pN1	131	18.32%	43	14.43%	84	22.70%	4	8.51%
pN2	113	15.80%	62	20.81%	46	12.43%	9	19.15%
Stage								
I	262	36.64%	114	38.26%	131	35.41%	15	31.91%
II	249	34.83%	88	29.53%	140	37.84%	16	34.04%
III	198	27.69%	91	30.54%	98	26.49%	15	31.91%
IV	6	0.84%	5	1.68%	1	0.27%	1	2.13%
Smoking status								
Neg.	104	14.55%	52	17.45%	38	10.27%	4	8.51%
Pos.	611	85.45%	246	82.55%	332	89.73%	43	91.49%

NSCLC—non-small cell lung cancer; AC—adenocarcinoma; SCC—squamous cell carcinoma; LCC–large cell carcinomas.

## Data Availability

The raw data and the analytic methods will be made available to other researchers upon reasonable request for the purpose of reproducing the results in their own laboratories. To access the protocols or datasets, contact katarzyna.ratajczak-wielgomas@umw.edu.pl.
